# Stirling Refrigerating Machine Modeling Using Schmidt and Finite Physical Dimensions Thermodynamic Models: A Comparison with Experiments

**DOI:** 10.3390/e23030368

**Published:** 2021-03-19

**Authors:** Cătălina Dobre, Lavinia Grosu, Alexandru Dobrovicescu, Georgiana Chişiu, Mihaela Constantin

**Affiliations:** 1 Department of Engineering Thermodynamics, Engines, Thermal and Refrigeration Equipment, University Politehnica of Bucharest, Splaiul Independenței 313, 060042 Bucharest, Romania; catalina.dobre@upb.ro (C.D.); adobrovicescu@yahoo.com (A.D.); 2 Laboratory of Energy, Mechanics and Electromagnetic, Paris West Nanterre La Défense University, 50, Rue de Sèvres, 92410 Ville d’Avray, France; mgrosu@parisnanterre.fr; 3 Department of Machine Elements and Tribology, University Politehnica of Bucharest, Splaiul Independenței 313, 060042 Bucharest, Romania; georgiana.chisiu@upb.ro

**Keywords:** Stirling refrigerator, thermodynamic analysis, numerical model, imperfect regeneration

## Abstract

The purpose of the study is to show that two simple models that take into account only the irreversibility due to temperature difference in the heat exchangers and imperfect regeneration are able to indicate refrigerating machine behavior. In the present paper, the finite physical dimensions thermodynamics (FPDT) method and 0-D modeling using the Schmidt model with imperfect regeneration were applied in the study of a β type Stirling refrigeration machine.The 0-D modeling is improved by including the irreversibility caused by imperfect regeneration and the finite temperature difference between the gas and the heat exchangers wall. A flowchart of the Stirling refrigerator exergy balance is presented to show the internal and external irreversibilities. It is found that the irreversibility at the regenerator level is more important than that at the heat exchangers level. The energies exchanged by the working gas are expressed according to the practical parameters, necessary for the engineer during the entire project. The results of the two thermodynamic models are presented in comparison with the experimental results, which leads to validation of the proposed FPDT model for the functional and constructive parameters of the studied refrigerating machine.

## 1. Introduction

The continued growth in the demand for refrigeration in almost all parts of the world and global warming due to the consumption of chlorofluorocarbon (HCFC) refrigerant has led the engineering community to seek applications for vapor-compression refrigeration. The Stirling refrigeration cycle is an important cycle model in the research and manufacture of refrigerators. The Stirling cycle machine is an alternative that could work with an environmentally friendly cooling fluid [1].

The Stirling cycle refrigerating machine was first developed in 1832 [2] but the system was first practically made in 1862, when Alexander Kirk built and patented a closed-cycle refrigerator. In 1971, Beale stated that by reversing the cycle, the Stirling cycle could be used for both work production and refrigeration purposes [3].

The Stirling reversible refrigeration cycle, for the same temperature range under perfect regenerative conditions [4], has the same coefficient of performance as the Carnot reversible refrigeration cycle according to classical thermodynamics.

The Stirling refrigerator is composed of two chambers with variable volume (expansion and compression) physically separated from the regenerator and with different temperatures. The presence of the regenerator (an economizer) qualifies the Stirling cycle machine as a regenerative machine.

According to the classical theory of thermodynamics, the performance of a Stirling cycle machine is a function of pressure, the ratio between temperature, speed and phase angle, fluid type, the efficiency of heat exchangers and volume [1].

Various thermodynamic models of Stirling machine operations have been proposed in the literature, with various assumptions. Schmidt developed the first performance analysis of the Stirling machine in 1871 and this proved to be an effective aspect of its design [5]. After Curzon and Ahlborn [6] studied the Carnot direct cycle using finite heat transfer, models were developed using finite time thermodynamics (FTT) [7], finite physical dimensions thermodynamics (FPDT) [8,9], and finite speed thermodynamics (FST) [10], which have been applied to several types of machines, including Stirling machines.

A finite time heat transfer analysis [7] was performed in 1998 for an air refrigeration cycle with non-isentropic compression and expansion. The relation between the coefficient of performance (COP) and the cooling load with the pressure ratio was obtained.

Petrescu et al. [10] developed an analytical model for estimating the performance of a Stirling engine based on the first and second laws of thermodynamics, called finite speed thermodynamics (FST). The model [11] directly connects the irreversibilities, and the flow and mechanical friction are taken into account.

Chen [12] developed an irreversible cycle model in order to predict the performance and input power required for a Stirling refrigerator optimized to a specified cooling capacity.

A β-type Stirling cycle refrigeration machine was mathematically designed and experimentally tested in [13]. Those authors studied the types of working fluids, the effect of the phase difference of the piston and the displacer on the refrigeration performance, the effect of parameters such as the ratio between the expansion volume and the compression volume and the dead volume ratio.

For the Stirling cycle refrigerator, Ataer and Karabulut [14] performed an analysis on the thermodynamic control volume subjected to periodic mass flow and evaluated the performed activity, instantaneous pressure and coefficient of performance.

A nonlinear mathematical model was developed for an air-filled Stirling alpha refrigerator by incorporating thermodynamics, wall heat transfer and fluid resistance in the regenerator. Different variables were also determined for both workspaces [15].

The effects of different parameters on the cooling performance of a Stirling cryocooler were also investigated [16]. It was found that the highest work loss was due to mechanical friction loss and the highest heat loss was due to conduction loss.

The performance of a β-type Stirling refrigeration machine with a regenerative displacer was studied by Hachem et al. [17,18], considering the complex phenomena related to the mechanics of compressible fluids, heat transfer and thermodynamics for energy analysis. An experimental validation with a focus on evaluating the effect of geometric parameters, such as the expansion space, the volume of the dead space and the compression of the swept volume was performed in [18]. The authors analyzed and optimized the parameters of the regenerator regarding the performance of the refrigerator. The various losses associated with the Stirling refrigerator that directly affect its cooling performance were evaluated. They described these losses as a function of the length and diameter of the regenerator.

Given the imperfection of the practical regenerator, researchers [19,20] have developed many thermodynamic models of Stirling engines using finite time thermodynamics (FTT). Based on finite speed thermodynamics (FST), Petrescu et al. [21] developed a method for calculating the coefficient that characterizes regenerative loss in a Stirling machine, based on the first law for processes with finite speed. Based on isothermal theory, Kongtragool and Formosa [22] studied the effect of regenerative efficiency and dead volume on a Stirling engine with an imperfect regenerator.

In the present paper, a finite physical dimensions thermodynamic (FPDT) method and 0-D modeling (isothermal analysis) using the Schmidt model (second order) with imperfect regeneration were applied in the study of a β-type Stirling refrigeration machine, with academic use and benefit.

The findings of Feidt et al. [23] show that the most significant reduction in performance is due to the non-adiabatic regenerator. The isotherm model in this paper is improved by including the irreversibility caused by imperfect regeneration and the finite temperature difference between the gas and the wall of the heat exchangers (cold and hot). The numerical model describes the evolution of instantaneous variables (pressure, volume, mass, changed energy, irreversibility) depending on the rotation angle of the shaft.

The FPDT model [16,24] is based on the irreversible thermodynamics approach, which is an old approach, but has had some improvements and engineering adjustments, which were the aims of recent papers by Grosu et al. [25,26,27].

The results obtained after applying the two models of thermodynamic analysis justify a more realistic evaluation of the FPDT model by reporting the experimental results. In this context, in order to identify the limitations of the isothermal model, this research was completed with an exergetic analysis of a β-type Stirling refrigerator that allows the development of a system of equations that describes the processes that take place at each element of the machine. The purpose of developing this method of thermodynamic analysis was to establish the value of irreversible losses in the actual cycle of the refrigeration machine and determine the cycle component to be improved in order to reduce the degree of irreversibility of the cycle.

## 2. Materials and Method

### 2.1. Description of Experimental Installation

A β-type Stirling refrigerator consisting of an arrangement with a displacer, power piston and regenerator in line was analyzed. A cylinder of highly resilient glass is surrounded by a water jacket in which a stream of water is the hot tank of the system operating as a refrigeration machine. The displacer forces the gas (air) to pass from the bottom space to the top space of the cylinder and vice versa. It also has an extremely conductive material, which is used for heat storage/release, thus acting as a regenerator, in order to improve the efficiency. The two pistons perform an alternating reciprocating motion with an angle of 110°.

**Figure 1:**
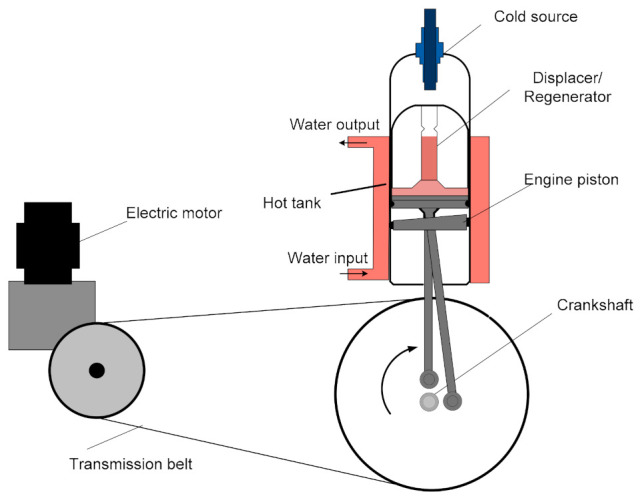
Experimental device using a β-type Stirling refrigerating machine.

The experimental device can function as an engine by providing mechanical work, or as a refrigerating machine (reverse cycle) by using an electric motor that drives the machine shaft [28]. The configuration of the Stirling refrigerator proposed for this study is shown in Figure 1. At the top of the cylinder is a thermocouple that allows temperature measurement and an electrical resistance that helps to determine the refrigerating power through a compensation method.

### 2.2. Application of Schmidt Method with Imperfect Regeneration in the Study of a β-Type Stirling Refrigerating Machine

The isothermal analysis (Schmidt method) takes into account the external and internal irreversibility of the machine and the kinematics of the pistons. In addition, the uneven distribution of time and space of the working fluid in the machine is taken into consideration by dividing the refrigerator into three volumes associated with a characteristic temperature. The assumptions that Schmidt considered in his analysis included the following: (a) the fluid in the compression volume of the refrigerating machine and the cold exchanger is always kept at a constant temperature, and the fluid temperature in the expansion volume and the hot-end heat exchanger is constant; (b) the surface temperature of the cylinder and the piston is constant; (c) the mass of the fluid is constant, which implies that there is no leakage and the same instantaneous pressure on the whole machine; (d) an ideal gas is used as a working fluid (perfect gas equation of state is applied); (e) there is harmonic/sinusoidal movement of the pistons (idealized crankshaft); and (f) the speed of working fluid within the machine is constant. The hypothesis of energy loss independence is used in this method [28].

In practice, this hypothesis, according to which the gas behaves isothermally in the expansion and compression spaces, is not true at high speeds. At high speeds, compression and expansion processes are closer to adiabatic processes [5].

Given the constructive peculiarities of the machine studied in this paper, it was operated at very low speeds and in order to model the Stirling refrigeration machine with some realism, the isothermal model was adapted.

**Figure 2:**
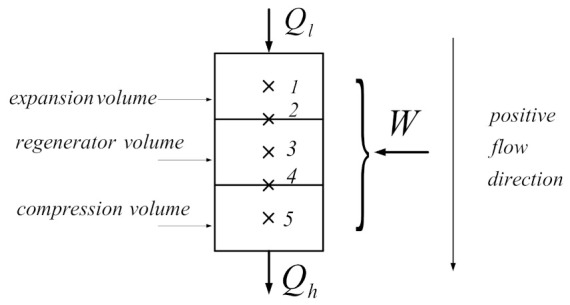
Representation of three volumes of machine and their boundaries.

The Schmidt method is based on dividing the refrigeration machine into three spaces: the expansion volume, the regenerator volume, and the compression volume. (Figure 2). Each part is considered a control volume, to which the laws of energy and mass conservation are applied.

**Figure 3:**
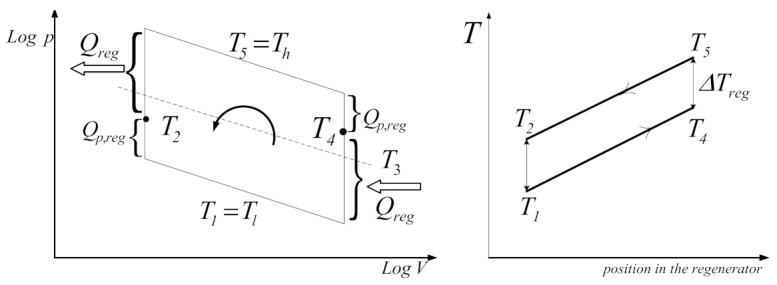
Temperature gradient in refrigerator regenerator.

According to the assumption, the gas temperature history will remain the same and part of the regenerated heat loss will be continuously compensated by a heat supplement *Q_p,reg_* provided by the source, as each cycle is driven by imperfect regeneration (Figure 3). Using refrigerator geometry, the volumes of compression and expansion spaces can be expressed according to the instantaneous positions of the pistons [29].

The following equation is used to determine the instantaneous volume of the compression space (hot):

(1) $$V_{C}=\frac{V_{C0}}{2}\left[1-\cos\phi \right]+V_{mC},$$

where ϕ is the idealized crankshaft rotation angle and *V_C_*_0_ is the swept compression volume; this is the displacer swept volume in the case of β-type Stirling machines.

The instantaneous volume of the expansion space (cold) is a combination of several volumes and can be determined as:

(2) $$V_{E}=\left\{\frac{V_{C0}}{2}\cdot \left[1+\cos(\phi )\right]+\frac{V_{E0}}{2}\left[1-\cos\left(\phi -\phi_{0}\right)\right]-V_{0l}\right\}+V_{mE},$$

where $\phi_{0}$ is the phase lag angle of the piston movements and *V_E0_* is the swept expansion volume. $V_{0l}$ is the overlapping volume in the case of a β-type Stirling machine and is due to the intrusion of the displacer piston into the working piston swept volume.

The dead volumes $V_{mE}$ and $V_{mC}$ on the heat exchangers are also taken into account.

To evaluate the mass of fluid in each volume, the state equation of the perfect gases is used. The instantaneous pressure is assumed to be uniform in the machine and its variation can be determined by using the mass balance:

(3) $$p=\frac{mR}{\frac{V_{h}}{T_{h}}+\frac{V_{reg}}{T_{r}}+\frac{V_{l}}{T_{l}}}$$

The elementary masses of each volume are calculated with:

(4) $$\left\{\begin{array}{l} dm_{l}=\frac{pdV_{l}+V_{l}dp}{RT_{1}}=dm_{1}\\ dm_{h}=\frac{pdV_{h}+V_{h}dp}{RT_{5}}=dm_{5}\\ dm_{reg}=m_{reg}\frac{dp}{p}=dm_{5} \end{array}\right.$$

Considering the mass flow direction on the interface, the interface temperatures can be expressed as follows:

$dm_{2}=-dm_{l}$ if $dm_{2}<0$, then $T_{2}=T_{1}+\Delta T_{reg}$, otherwise, $T_{2}=T_{1}$;

$dm_{4}=-dm_{h}$ if $dm_{4}<0$, then $T_{4}=T_{5}$, otherwise, $T_{4}=T_{5}-\Delta T_{reg}$.

While differentiating Equation (3) and considering that the temperatures are constant, *dp* is obtained in the following form:

(5) $$dp=\frac{-p\left(\frac{dV_{l}}{T_{1}}+\frac{dV_{h}}{T_{5}}\right)}{\frac{V_{l}}{T_{1}}+\frac{V_{reg}}{T_{3}}+\frac{V_{h}}{T_{5}}}$$

The internal irreversibility of the studied Stirling cycle is assumed to be due to the imperfect regeneration. The regenerator/displacer reciprocating movement forces the air of the cooling space toward the heating space and conversely: it is also useful to store and release the heat exchanged with the regenerator material during this transfer (Figure 3). The difference is the temperature gap on the regenerator Δ*T_reg_*, assumed to be constant on the whole length of the regenerator [28]. Therefore, in the case of the Stirling refrigerator, the regenerator efficiency is defined by:

(6) $$\eta_{reg}=\frac{T_{5}-T_{4}}{T_{5}-T_{1}}=\frac{T_{2}-T_{1}}{T_{5}-T_{1}}=\frac{\Delta T_{reg}^{}}{T_{5}-T_{1}}$$

Thus:

(7) $$\begin{array}{l} T_{5}=T_{4}+\eta_{reg}\left(T_{5}-T_{1}\right)\\ T_{2}=T_{1}+\eta_{reg}\left(T_{5}-T_{1}\right) \end{array}$$

In the regenerator, the changed work is zero and the average temperature is supposed to be constant $T_{reg}$. The regenerator temperature, $T_{reg}=T_{3}$, is a logarithmic average of cold and hot space ($V_{C}$ and $V_{E}$) temperatures:

(8) $$T_{3}=T_{reg}=\frac{T_{h}-T_{l}}{\ln\frac{T_{h}}{T_{l}}}=\frac{T_{5}-T_{1}}{\ln\frac{T_{5}}{T_{1}}}$$

The quantity of heat changed at the level of the three volumes is obtained starting from the energy conservation equation applied to each volume:

(9) $$\left\{\begin{array}{l} \delta Q_{l}=\left(\frac{c_{v}}{R}+1\right)pdV_{l}+\frac{c_{v}}{R}V_{l}dp+c_{p}T_{2}dm_{2}\\ \delta Q_{reg}=V_{reg}\frac{c_{v}}{R}dp+c_{p}\left(T_{4}dm_{4}-T_{2}dm_{2}\right)\\ \delta Q_{h}=\left(\frac{c_{v}}{R}+1\right)pdV_{h}+\frac{c_{v}}{R}V_{h}dp-c_{p}T_{4}dm_{4} \end{array}\right.$$

The elementary mechanical work in the compression $\delta W_{h}=-pdV_{h}$ and expansion $\delta W_{l}=-pdV_{l}$ spaces allow, after integration, calculation of the mechanical work consumed in a cycle:

(10) $$W=W_{l}+W_{h}$$

The temperatures of the expansion and compression spaces are determined starting with heat flow rates and from the global heat transfer coefficients, experimentally obtained:

(11) $$\left|{\dot{Q}}_{h}\right|=hA_{h}\left(T_{h}-T_{wh}\right)\to T_{h}=T_{wh}+\frac{\left|{\dot{Q}}_{h}\right|}{hA_{h}}$$

(12) $${\dot{Q}}_{l}=hA_{l}\left(T_{wl}-T_{l}\right)\to T_{l}=T_{wl}-\frac{{\dot{Q}}_{l}}{hA_{l}}$$

The equations presented above were solved using the Simulink simulation tool. In order to improve the obtained results based on the isothermal method (Schmidt) by taking temperature levels into account, an exergetic analysis is required for the β-type Stirling refrigerating machine.

### 2.3. Application of Exergetic Method in the Study of β-Type Stirling Refrigerating Machine

#### 2.3.1. Exergetic Analysis Applied in the Study of the β-Type Stirling Refrigeration Machine

The simple and fast processing of the energy balance and exergetic balance equations leads to obtaining the classic exergetic balance equations customized on the reversed cycle (refrigeration installation), written at the consumer level:

(13) $$\left|\dot{W}\right|=\left|\dot{E}x_{Q_{wl}}^{T_{wl}}\right|+\left|\dot{E}x_{Q_{h}}^{T_{h}}\right|+\dot{E}x_{l}^{D}+\dot{E}x_{reg}^{D},$$

where:

$\dot{E}x_{l}^{D}$ is exergy destruction due to heat transfer at the finite difference in the cooler; $\dot{E}x_{reg}^{D}$ is the exergy destruction in the regenerator and $\left|\dot{E}x_{Q_{h}}^{T_{h}}\right|$ is loss of exergy with heat discharged into the environment.

**Figure 4:**
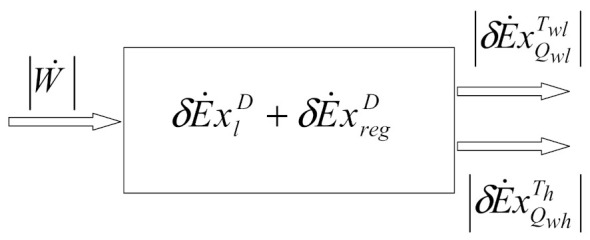
Exergy balance for β-type Stirling refrigerating machine.

The schematic of the exergy balance for the Stirling refrigerating machine is presented in Figure 4.

For the calculation of terms in Equation (13), in the following, the exergetic balances are established at the level of each element of the Stirling machine, depending on the kinematics of the pistons.

The exergetic balance in differential form is applied for each heat exchanger:

(14) $$dEx=\delta Ex_{Q}^{T}+\delta W+p_{0}dV+ex_{i}^{f}dm_{i}-ex_{e}^{f}dm_{e}-T_{0}\delta \Pi ,$$

where $\delta Ex_{Q}^{T}$ is the exergy of heat at the temperature T of the system.

Then we obtain:

(15) $$dEx_{reg}=\left(1-\frac{T_{0}}{T_{reg}}\right)\delta Q_{reg}+\delta W_{reg}+p_{0}dV_{reg}+ex_{2}^{f}dm_{2}-ex_{4}^{f}dm_{4}-T_{0}\delta \Pi_{reg}$$

(16) $$dEx_{l}=\left(1-\frac{T_{0}}{T_{l}}\right)\delta Q_{l}+\delta W_{l}+p_{0}dV_{l}-ex_{2}^{f}dm_{2}$$

(17) $$dEx_{h}=\left(1-\frac{T_{0}}{T_{h}}\right)\delta Q_{h}+\delta W_{h}+p_{0}dV_{h}+ex_{4}^{f}dm_{4}$$

For a cycle, the balance can be written as follows:

(18) $$dEx_{l}+dEx_{reg}+dEx_{h}=0$$

Using the equations presented above, $\delta \Pi_{reg}$ and $\Pi_{reg}$ can also be calculated.

#### 2.3.2. Study of Heat Exchangers (Compression and Expansion Volume) and Calculation of Exergy Destroyed due to Temperature Differences

##### Cold-End Heat Exchanger Study

**Figure 5:**
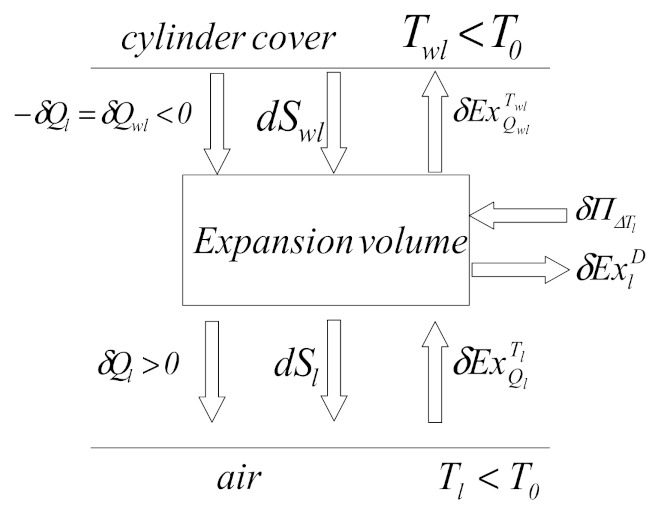
Exergetic and entropic functional diagram of expansion volume.

A functional diagram of the expansion volume (Figure 5) shows the entropies and exergies exchanged by the air in the expansion volume of the refrigeration machine with a cold source (cylinder head).

The exergetic balance allows the determination of exergy lost due to temperature differences between the expansion volume and the cylinder head:

(19) $$\left|\delta Ex_{Q_{l}}^{T_{l}}\right|=\delta Ex_{l}^{D}+\delta Ex_{Q_{wl}}^{T_{wl}},$$

where:

(20) $$\delta Ex_{Q_{l}}^{T_{l}}=\left(1-\frac{T_{0}}{T_{l}}\right)\delta Q_{l}<0$$

represents the exergy of heat $\delta Q_{l}$ at temperature $T_{l}$.

The exergy of heat $\delta Q_{wl}$ at temperature $T_{wl}$ is given by the equation:

(21) $$\delta Ex_{Q_{wl}}^{T_{wl}}=\left(1-\frac{T_{0}}{T_{wl}}\right)\delta Q_{wl}>0,$$

where $\delta Ex_{Q_{wl}}^{T_{wl}}$ is the useful effect of the refrigeration machine in exergetic terms.

Replacing relations (20) and (21) in Equation (19) of the exegetical balance, the destroyed exergy at the level of the cold exchanger results in:

(22) $$\delta Ex_{l}^{D}=\left|\delta Ex_{Q_{l}}^{T_{l}}\right|-\delta Ex_{Q_{wl}}^{T_{wl}}=\left(\frac{T_{0}}{T_{l}}-1\right)\delta Q_{l}-\left(1-\frac{T_{0}}{T_{wl}}\right)\left(-\delta Q_{l}\right)$$

By grouping the terms, we can obtain:

(23) $$\delta Ex_{l}^{D}=T_{0}\delta Q_{l}\left(\frac{1}{T_{l}}-\frac{1}{T_{wl}}\right)$$

The destroyed exergy flow rate is calculated by integrating relation (23) over the entire cycle of the refrigeration machine during a complete rotation of the shaft:

(24) $$\dot{E}x_{l}^{D}=n\oint \delta Ex_{l}^{D}$$

The exergetic efficiency of the cold exchanger is:

(25) $$\eta_{ex_{l}}=\frac{\dot{E}x_{Q_{wl}}^{T_{wl}}}{\left|\dot{E}x_{Q_{l}}^{T_{l}}\right|},$$

and the dissipation coefficient is:

(26) $$\zeta_{l}=\frac{\dot{E}x_{l}^{D}}{\left|\dot{E}x_{Q_{l}}^{T_{l}}\right|}$$

##### Hot-End Heat Exchanger Study

The gas temperature of the compression volume is higher than the ambient temperature, and as the gas is cooled, its exergy will decrease, so the exergy flow rate will have the same direction as the heat transfer.

**Figure 6:**
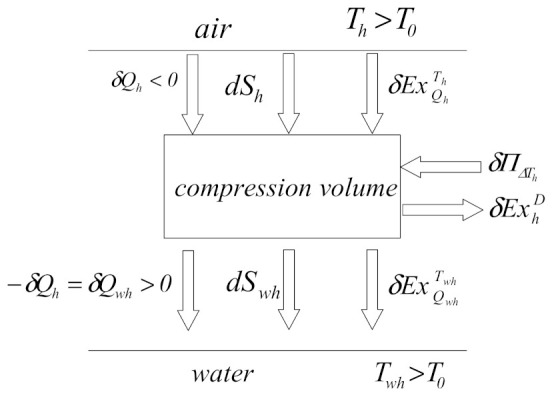
Exergetic and entropic functional diagram of compression volume.

A functional diagram of the compression volume shows the exchanged air exergy with a cold source (Figure 6).

The exergetic balance of the compression chamber can be written as follows:

(27) $$\left|\delta Ex_{Q_{h}}^{T_{h}}\right|=\delta Ex_{h}^{D}+\delta Ex_{Q_{wh}}^{T_{wh}},$$

where the exergy ofheat $\delta Q_{h}$ at temperature $T_{h}$ is:

(28) $$\delta Ex_{Q_{h}}^{T_{h}}=\left(1-\frac{T_{0}}{T_{h}}\right)\delta Q_{h}<0$$

The exergy of heat $\delta Q_{wh}$ at temperature $T_{wh}$ is:

(29) $$\delta Ex_{Q_{wh}}^{T_{wh}}=\left(1-\frac{T_{0}}{T_{wh}}\right)\delta Q_{wh}>0$$

The exergy lost at the hot-end heat exchanger due to the temperature difference between the compression room and the hot source can be calculated as follows:

(30) $$\delta Ex_{h}^{D}=\left|\delta Ex_{Q_{h}}^{T_{h}}\right|-\delta Ex_{Q_{wh}}^{T_{wh}}=-\left(1-\frac{T_{0}}{T_{h}}\right)\delta Q_{h}+\left(1-\frac{T_{0}}{T_{wh}}\right)\delta Q_{h}$$

(31) $$\delta Ex_{h}^{D}=T_{0}\delta Q_{h}\left(\frac{1}{T_{h}}-\frac{1}{T_{wh}}\right),$$

and the exergy destroyed at the hot-end heat exchanger level is:

(32) $$\dot{E}x_{h}^{D}=n\oint \delta Ex_{h}^{D}$$

The exergetic efficiency of the hot-end heat exchanger can be calculated as:

(33) $$\eta_{ex_{h}}=\frac{\dot{E}x_{Q_{wh}}^{T_{wh}}}{\left|\dot{E}x_{Q_{h}}^{T_{h}}\right|},$$

and its dissipation coefficient as:

(34) $$\varsigma_{h}=\frac{\dot{E}x_{h}^{D}}{\left|\dot{E}x_{Q_{h}}^{T_{h}}\right|}$$

### 2.4. Application of TDFF in the Study of the β-Type Stirling Refrigerating Machine

Finite physical dimensions thermodynamics (FPDT) [13,14,15,16,17] is a method that regroups the techniques of thermodynamics in finite time, speed and geometric dimensions. This method introduces the exo-irreversibilities due to the finite heat transfer between sources (hot source, cold source, regenerator) and the working fluid. In addition, it considers the constraints faced by engineers. Using classical thermodynamics, it has been shown that machines with or without heat generation operating after cycles similar to the Carnot cycle can be described by using physical parameters such as *p*_max_, *V*_max_, $T_{h}$, and $T_{l}$ as reference parameters. It is essential to consider the rotation speed as the main variable, because heat and mass transfer are dependent in a straightforward manner on speed and naturally must be expressed accordingly.

**Figure 7:**
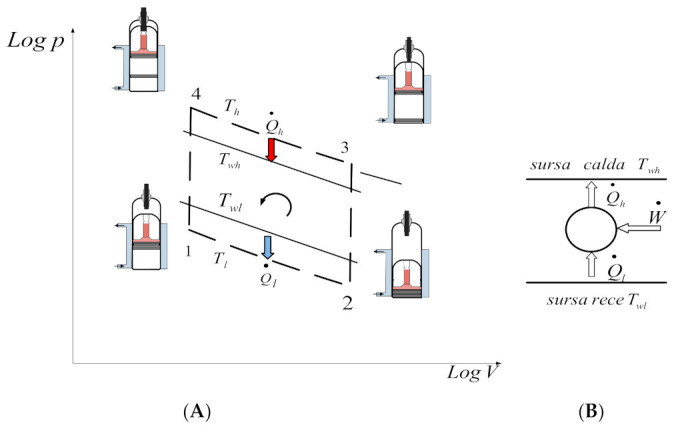
Exo-irreversible reversed Stirling cycle. (**A**) Logp-LogV diagram in the range limit of $p_{\max}$, $V_{\max}$, $T_{l}$ and $T_{l}$; (**B**) energy balance scheme.

In the following, the FPDT method is applied in the study of the exo-irreversible reversed Stirling cycle with imperfect regeneration, represented in Figure 7.

The main hypothesis of this method of thermodynamic analysis is that the reheater and the compression space are at the same temperature, as are the cooler and the expansion space.

It is also considered that the gas that is used is a perfect gas and its total mass is supposed to be transferred entirely from the hot volume to the cold volume and vice versa (neglecting the dead volume), remaining constant throughout the experiment (it is considered a closed thermodynamic system).

The energies transferred in the cycle are given by the following relations.

The heat given to the hot tank (water) by the working gas at temperature $T_{h}$, in the case of perfect regeneration, in absolute value, is:

(35) $$\left|Q_{h.rev}\right|=\left|Q_{34}\right|=p_{\max}V_{\max}\frac{\ln\varepsilon }{\varepsilon }=E_{\varepsilon },$$

where $E_{\varepsilon }$ is the reference energy of the FPDT model.

The heat taken from the cold tank by the working gas at temperature $T_{l}$, in the case of perfect regeneration, is:

(36) $$Q_{l.rev}=Q_{12}=p_{\max}V_{\max}\frac{\ln\varepsilon }{\varepsilon }\frac{T_{l}}{T_{h}}=E_{\varepsilon }\frac{T_{l}}{T_{h}}$$

The heat exchanged with the regenerator (stored and detached) during an isochoric transformation is:

(37) $$Q_{reg}=mc_{v}\left(T_{h}-T_{l}\right)=m\frac{R}{\gamma -1}T_{h}\left(1-\frac{T_{l}}{T_{h}}\right)$$

The regeneration efficiency is described by the relation:

(38) $$\eta_{reg}=\frac{Q_{reg}-Q_{p,reg}}{Q_{reg}},$$

where $Q_{p,reg}$ is the amount of heat to be added to that received by the hot source and given by the cold source ($Q_{p,reg}>0$).

It follows that:

(39) $$Q_{p,reg}=\left(1-\eta_{reg}\right)Q_{reg}=E_{\varepsilon }k\left(1-\frac{T_{l}}{T_{h}}\right)$$

The notation k is used to define the regenerative loss factor:

(40) $$k=\frac{1-\eta_{reg}}{\ln\varepsilon \left(\gamma -1\right)}$$

Heat quantities change in the case of imperfect regeneration:

(41) $$\left|Q_{h}\right|=\left|Q_{34}\right|-Q_{p,reg}=E_{\varepsilon }\left[1-k\left(1-\frac{T_{l}}{T_{h}}\right)\right]$$

(42) $$Q_{l}=Q_{12}-Q_{p,reg}=E_{\varepsilon }\left[\frac{T_{l}}{T_{h}}-k\left(1-\frac{T_{l}}{T_{h}}\right)\right]$$

The mechanical work consumed per cycle in absolute value results in:

(43) $$W=\left|Q_{h}\right|-Q_{l}$$

It should be mentioned here that the mechanical work consumed in a cycle is independent of the regeneration efficiency $\eta_{reg}$.

Using Equation (41), the balance of heat flows at the hot/cold source are obtained:

(44) $$\left|{\dot{Q}}_{h}\right|=n\left|Q_{h}\right|=nE_{\varepsilon }\left[1-k\left(1-\frac{T_{l}}{T_{h}}\right)\right]=K_{h}\left(T_{h}-T_{wh}\right)$$

(45) $${\dot{Q}}_{l}=nQ_{l}=nE_{\varepsilon }\left[\frac{T_{l}}{T_{h}}-k\left(1-\frac{T_{l}}{T_{h}}\right)\right]=K_{l}\left(T_{wl}-T_{l}\right)$$

The COP performance coefficient of the Stirling refrigeration machine can be determined with the equation:

(46) $$COP=\frac{Q_{l}}{W}$$

## 3. Results and Discussions

### 3.1. Experimental Results

The considered experimental device is a reversible thermal machine (motor and/or receiver) that operates between two heat sources at constant temperature. It works according to the Stirling cycle.

The Stirling refrigerator analyzed is equipped with several sensors: thermocouples, position sensors, pressure sensors, instantaneous position piston sensor, and a device composed of photodiodes and a drilled disk to measure the speed of rotation of the flywheel. The rotation speed *n* of the electric motor can be varied by means of a control and adjustment device.

The refrigerating power of the analyzed cooling system is estimated by a compensation method by means of a small electric resistance placed inside the cold room (located at the top of the cylinder). In this way, the air at the top of the cylinder cools and heats at the same time. A temperature equal to the ambient temperature can be set inside the cylinder in order to limit the losses through the cylinder wall. In this way, the refrigeration power that corresponds to the heat flow rate taken from the cylinder head of the refrigeration machine is determined with the relation:

(47) $${\dot{Q}}_{l}=UI$$

where U is the voltage (V) and I is the intensity of the electric current (A) corresponding to the electrical compensation resistance.

The thermal conductivity of the cold tank wall can be determined starting from the relation:

(48) $${\dot{Q}}_{l}=K_{l}\Delta T_{l},$$

where

${\dot{Q}}_{l}$ is the refrigerating power of the cooling system, determined by the compensation method, (W); $\Delta T_{l}=T_{l}-T_{wl}$, with $T_{l}$ representing the gas temperature measured inside the cold volume (K); and $T_{wl}$ is the wall temperature of the cold volume, measured with a thermocouple (K).

From the relation of thermal conductivity, we can calculate the global heat exchange coefficient:

(49) $$h=\frac{K_{l}}{A_{l}},$$

where $A_{l}$ is the contact area of the cold exchanger (upper part of the cylinder) (m^2^).

**Figure 8:**
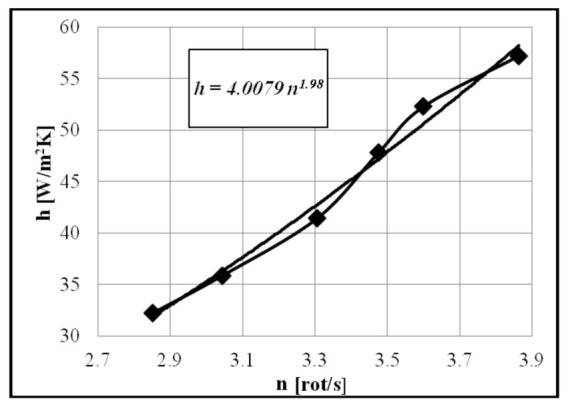
Variation of overall heat transfer coefficient h depending on rotational speed n.

The parameters $T_{l}$, $T_{wl}$ and ${\dot{Q}}_{l}$ are experimentally determined for several operating modes. The heat transfer coefficient *h* is calculated for each speed; as expected, and according to the existing data in the literature [30], the overall heat transfer coefficient *h* increases with increasing rotational speed *n* (Figure 8).

The variation of cooling water temperature and the circulating water flow rate allow us to calculate the power yielded to the water, with the relation:

(50) $${\dot{Q}}_{h}={\dot{m}}_{w}c_{w}\Delta T_{w},$$

where

$c_{w}$ is water-specific heat (J/kgK); and $\Delta T_{w}=T_{w}^{e}-T_{w}^{i}$, with $T_{w}^{e}$ representing the output water temperature (K) and $T_{w}^{i}$ the input water temperature.

It can be concluded from Table 1 that as the rotation speed *n* increases, the temperature of the cold gas increases and the temperature of the hot gas decreases. The difference between the two temperatures decreases, which implies increased COP of the refrigerating machine with increased speed.

### 3.2. Thermodynamic Analysis and Analytical Simulation Results

Using geometric and functional parameters (Table 2) measured or determined by the acquisition program (CassyLab) and using the calculation algorithm, the following developments are obtained depending on the engine rotation speed.

The rotation speed of cooling varied between 2.5 and 3.86 rot/s during the tests, when air was used as working fluid.Any fluid change is not appropriate, as this system has academic use and benefit. The pressure load is 1 bar and should remain, so this refrigerator requires a small amount of mechanical power. The two pistons perform alternating reciprocating motion with an angle of 110°.

The initial data of the simulated point (n = 3.86 rot/s) are listed in Table 3.

Comparable values were obtained for the exchanged heat flow rates and the mechanical power required to operate the refrigerating machine (Table 4), calculated by processing the experimental data with the two calculation models, 0-D and FPDT.

The differences obtained between the experimentally processed values and those obtained using of the Schmidt analysis model with imperfect heat regeneration (0-D model) and FPDT model are also reflected in the exergetic calculation of the exchangers. (Table 5 and Table 6).

(a) Cold-End Heat Exchanger

(b) Hot-End Heat Exchanger

**Figure 9:**
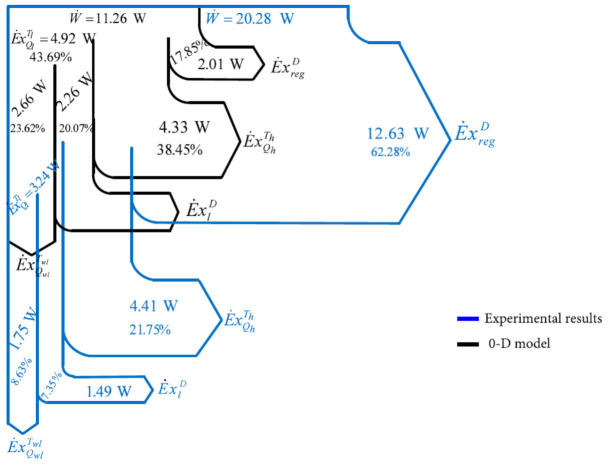
Flowchart of exergy balance equation.

A flowchart of the exergy balance equation [31] (Equation (27)) is presented in Figure 9.

The percentage share in the mechanical power input of each exergy current is presented in Figure 9 as well. Figure 9 shows the difference between the results obtained based on the experimental values presented by comparison with those obtained using the 0-D model. The irreversibility at the level of the regenerator is more important than that at the level of the heat exchangers.

The values obtained when applying the global exergetic efficiency formula $\left(\eta_{EX}=\frac{\dot{E}x_{Q_{wl}}^{T_{wl}}}{\dot{W}}\right)$ are shown in Table 7.

Table 8 compares the results of the two models of thermodynamic analysis and the experimentally obtained results for the Stirling machine.

The experimental COP of the refrigerating machine for a cold temperature of −22.45 °C is found to be 0.947. From the same cold temperature, the COP obtained after applying the 0-D model was found to be 2.57, with an error of 171.38%.

Applying the FPDT model returns a value of 0.905 for the COP. This shows that the simulation results approach the experimental results with an error of 4.43%.

In terms of evaluating the mechanical power input at the same speed, n = 3.86 rot/min (corresponding to the cold space air temperature *t_h_* = −22.45 °C), after numerical simulation the 0-D model returns a value of 11.26 W (error of 44.47%), while in the FPDT model, the mechanical power required for operating the Stirling refrigerator is 19.68 W, with an error of 2.95%.

In addition, and after comparing the values of the global exergetic efficiency (Table 7) obtained for the two proposed thermodynamic analysis methods, the 0-D model provides an $\eta_{EX}$ of 23.65 with an error of 174%, while the global exergetic efficiency calculated with the FPDT model is 8.23 (error of 4.63%).

## 4. Conclusions

A 0-D numerical model describing the evolution of variables (pressure, volume, mass, exchanged energy, irreversibility) as a function of the crankshaft angle is presented. The model uses the energy and exergy balance in a controlled volume, assuming a steady-state operation in the Stirling refrigerator, in order to obtain the overall irreversibility of the heat exchangers. External irreversibility is due to a finite temperature difference between gas and heat exchangers, while internal irreversibility is due to regenerative heat loss and entropy generation. It is found that the irreversibility at the level of the regenerator is more important than that at the level of the heat exchangers (Figure 9).

A flowchart of the exergy balance of the Stirling refrigerator is presented to show the internal and external irreversibilities (destroyed exergy flow). In the flow diagram (Figure 9), the exergy flows of the working gas with two reservoirs (heat from hot source and heat to cold sink) are shown at different temperatures, *T_h_* and *T_wh_* for the source and *T_l_* and *T_wl_* for the sink.

The study was completed by comparing the results obtained with the isothermal model and the FPDT model. The irreversibilities that FPDT model takes into account are exo-irreversibilities due to the finite heat transfer between the sources (hot source, cold source, regenerator) and the working fluid.

Regarding the evaluation of the mechanical power necessary for operating the refrigeration machine using the Schmidt isothermal model with imperfect regeneration, the difference between the experimental results and the results given by the thermodynamic model is justified by the fact that friction and aerodynamic losses are not taken into account in this model.

The results of the two thermodynamic models are presented in comparison with the experimental results, which leads to validation of the proposed FPDT model for the functional and constructive parameters of the studied refrigerating machine. It is found that the calculated values are very close to the experimental values, which validates the proposed analysis model for the β-type Stirling refrigerator. Therefore, the FPDT model proves to be a useful tool for analyzing the performance (COP and input power) of β-type Stirling refrigeration machines.

## Figures and Tables

**Table 1:** Centralization of experimental data obtained for the Stirling refrigerator.

*n* (rot/s)	*T_l_* (K)	*T_h_* (K)	Δ T (K)	${\dot{Q}}_{l}$ (W)	${\dot{Q}}_{h}$ (W)	${\dot{W}}_{\exp}$ (W)	*COP*_*exp*_ (–)
2.85	249	348.69	99.69	12.35	32.57	20.22	0.61
3.04	249.5	343.99	94.49	13.40	33.56	20.16	0.66
3.31	250.3	338.03	87.73	14.70	34.54	19.84	0.74
3.47	250.4	332.72	82.32	16.50	35.53	19.03	0.87
3.60	249	330.34	81.34	17.94	37.51	19.57	0.92
3.86	250.7	329.10	78.40	19.20	39.48	20.28	0.95

**Table 2:** Dimensional data of the actual engine.

$A_{h}$ (m^2^)	$A_{l}$ (m^2^)	$V_{\min}\cdot 10^{-4}$ (m^3^)	$V_{\max}\cdot 10^{-4}$ (m^3^)	$D_{p}=D_{d}$ (m)	$C_{p}=C_{d}$ (m)	$\varphi_{0}$ (°)
0.01885	0.03717	1.906	3.278	0.06	0.0484	110

**Table 3:** Initial conditions of a simulated point.

*p*_min_ = 70,000 Pa			*p*_max_ = 211,600 Pa		
*n* (rot/s)	*T_l_* (K)	*T_wl_* (K)	*T_h_* (K)	*T_wh_* (K)	*T_0_* (K)
3.86	250.7	268.5	329.1	295	293

**Table 4:** Analyzed heat flow rates.

	Experiment	0-D Model	0-D Error (%)	FPDT Model	FPDT Error (%)
${\dot{Q}}_{l}(W)$	19.21	29.17	51.92	17.79	7.39
$\left|{\dot{Q}}_{h}\right|(W)$	39.47	40.22	1.90	37.48	5.04
${\dot{W}}_{l}(W)$	20.28	11.26	44.47	19.68	2.95

**Table 5:** Exergetic calculation of cold-end heat exchanger.

	Experiment	0-D Model	0-D Error (%)	FPDT Model	FPDT Error (%)
$\left|\dot{E}x_{Q_{l}}^{T_{l}}\right|(W)$	3.24	4.92	51.85	3	7.41
$\dot{E}x_{Q_{wl}}^{T_{wl}}(W)$	1.75	2.66	52	1.62	7.43
$\dot{E}x_{l}^{D}(W)$	1.49	2.26	51.67	1.38	7.38
$\eta_{ex_{l}}(\%)$	54.01	54.06	0.09	54.08	0.13
$\zeta_{l}(\%)$	45.98	45.93	0.10	46	0.15

**Table 6:** Exergetic calculation of the hot-end heat exchanger.

	Experiment	0-D Model	0-D Error (%)	FPDT Model	FPDT Error (%)
$\left|\dot{E}x_{Q_{h}}^{T_{h}}\right|(W)$	4.33	4.41	1.85	4.11	5.08
$\dot{E}x_{Q_{wh}}^{T_{wh}}(W)$	0.27	0.27	0	0.25	7.40
$\dot{E}x_{h}^{D}(W)$	4.06	4.13	1.70	3.86	4.92
$\eta_{ex_{h}}(\%)$	6.23	6.12	1.76	6.18	0.80
$\zeta_{h}(\%)$	93.76	93.65	0.11	93.91	0.16

**Table 7:** Global exergetic efficiency values.

	Experiment	0-D Model	FPDT Model
$\eta_{EX}(\%)$	8.63	23.65	8.23

**Table 8:** Comparison of experimental and analytical results from analyzed methods.

n = 3.86 (rot/min)					
$COP_{\exp}$	${\dot{W}}_{\exp}$	$COP_{0-D}$	${\dot{W}}_{0-D}$	$COP_{FPDT}$	${\dot{W}}_{FPDT}$
(–)	(W)	(–)	(W)	(–)	(W)
0.947	20.280	2.570	11.260	0.905	19.68

## Data Availability

The data presented in this study are available on request from the corresponding author.

## Nomenclature

*A*	heat exchange surface, m^2^
c	specific heat, Jkg^−1^K^−1^
*C*	stroke of the piston, m
*D*	diameter of the piston, m
*Ex*	Exergy, J
${\dot{E}}_{x}$	exergy flow rate, W
*h*	convective heat transfer coefficient, Wm^−2^K^−1^
*I*	current, A
*k*	losses factor in regenerator,
*K*	heat exchanger conductance, WK^−1^
*m*	mass, kg
*n*	engine rotation speed, rot·s^−1^
*p*	pressure, Pa
*Q*	heat, J
$\dot{Q}$	heat transfer rate, W
*R*	gas constant, Jkg^−1^K^−1^
*S*	entropy, JK^−1^
*s*	specific entropy, Jkg^−1^K^−1^
*T*	temperature, K
*U*	voltage, V
*V*	volume, m^3^
*W*	work, J
$\dot{W}$	mechanical power, W
*Greek symbols*	
γ	adiabatic exponent, -
φ	rotation angle, °
φ_0_	phase lag angle, °
*ε*	volumetric compression ratio (V_max_/V_min_), -
η	efficiency, -
Π	entropy increase, JK^−1^
$\dot{\Pi }$	rate of entropy increase, WK^−1^
ξ	dissipation coefficient, -
*Subscripts*	
*C*	compression
*ε*	depending on *ε*
*ex*	exergetic
*E*	expansion
*d*	displacer
*h*	hot on working gas side
*l*	low on working gas side
*m*	dead
*max*	maximum
*min*	minimum
*p*	piston
*rev*	reversible
*reg*	regenerator
*v*	constant volume (specific heat)
*wl*	wall on source side
*wh*	wall on sink side
